# Genetic and epidemiological characteristics of the spread of human metapneumovirus in Bulgaria, 2023–2025

**DOI:** 10.3389/fpubh.2026.1834413

**Published:** 2026-05-13

**Authors:** I. Trifonova, N. Korsun, D. Pavlova, Zh. Getsova, N. Rukmanski, T. Valkov, P. Velikov, I. Ivanov, T. Dakov, D. Ivanov, Y. Uzunova, I. Christova

**Affiliations:** 1 Department of Virology, National Centre of Infectious and Parasitic Diseases, National Laboratory “Influenza and ARD”, Sofia, Bulgaria; 2 Center of Competence “ImmunoPathogen”, Sofia, Bulgaria; 3 Department for Infectious Diseases, Parasitology and Tropical Medicine, Medical University of Sofia, Sofia, Bulgaria; 4 Affiliate “Prof. Dr. Ivan Mitev” Vratsa, Medical University of Sofia, Sofia, Bulgaria; 5 University Hospital “Lozenetz”, Sofia, Bulgaria; 6 Infectious Disease Hospital “Prof. Ivan Kirov”, Sofia, Bulgaria

**Keywords:** human metapneumovirus, lower respiratory tract infections, next-generation sequencing, phylogenetic analysis, severe acute respiratory infections

## Abstract

**Introduction:**

Human metapneumovirus (hMPV) can cause severe respiratory illnesses such as bronchitis, bronchiolitis, and pneumonia, particularly in young children, older adults, and individuals with weakened immune systems. This study aimed to assess hMPV circulation patterns, including seasonal and demographic distribution, track genetic evolution and mutations in surface proteins, and explore associations with clinical severity.

**Methods:**

From early 2023 to November 2025, Bulgaria’s National Reference Laboratory tested nasopharyngeal samples from 6,958 patients with respiratory symptoms using an in-house multiplex real-time PCR method that detected 11 viruses. Positive hMPV samples were sequenced and analyzed phylogenetically.

**Results:**

A study conducted 3 years after the COVID-19 pandemic found that 1.4% (97 patients) tested positive for hMPV, with most being mono-infected (85.6%) and others co-infected, primarily with rhinovirus and adenovirus. The infection rate was highest in children under 5 (2.8%), especially infants and toddlers (2.2%), compared to 0.4% in those over 65 (*p* < 0.05). Common symptoms in 56% of hMPV-positive patients included cough (50%), fever (38%), and rhinitis (21.2%). Among 94 patients with respiratory involvement, 34% had upper respiratory tract infections (URTs), and 55% had lower respiratory tract infections (LRTs), with bronchiolitis (46%) and pneumonia (30.7%) being common. Hospitalized patients had a 19% rate of severe acute respiratory infection (SARI), higher than the 7.3% for other viruses. Sequencing revealed genotypes A and B in similar proportions, with subclade B2 being the most common at 41.3%. However, in 2025, subclade A2b2 represented 52% of the samples. The A2b2 subclade in children under 5 showed a higher incidence of lower respiratory complications (*p* < 0.05), while genotype B was linked to an increased risk of SARI at higher viral loads (*p* < 0.05).

**Conclusion:**

The findings confirm that hMPV is a significant respiratory pathogen for young children, showing substantial genetic diversity in Bulgaria, with lineage A2b2 becoming more prevalent in 2025. Host age and viral genetics may influence disease presentation. These results highlight the clinical burden of hMPV and emphasize the need for preventive strategies, including vaccine development for high-risk children.

## 1 Introduction

Following the COVID-19 pandemic, many countries implemented stringent anti-epidemic measures, which led to atypical surges in respiratory viral and bacterial infections after these measures were lifted during and after the COVID era (1, 2). For example, in 2024, China reported a significant increase in human metapneumovirus (hMPV) infections, raising public health concerns about the spike in respiratory pathogen morbidity (3). hMPV, a *Paramyxovirus* first identified in 2001, belongs to the genus Metapneumovirus within the family *Pneumoviridae* and is estimated to account for approximately 10–12% of respiratory infections in children (4).

hMPV can cause severe respiratory complications, such as bronchitis, bronchiolitis, and pneumonia (5). The risk of severe disease is particularly high among vulnerable populations, including young children, older adults, and individuals with compromised immune systems, such as those receiving immunosuppressive therapy or living with chronic diseases (6). In addition, hMPV infections can exacerbate symptoms in patients with asthma and chronic obstructive pulmonary disease (7). hMPV infections exhibit a seasonal pattern of occurrence, typically peaking in late winter and early spring in temperate regions, overlapping with other respiratory viruses such as respiratory syncytial virus (RSV) (8). Older studies reported low prevalence rates of hMPV infection: 0.2% in 2007, 4.3% in 2008, and 0.3% in 2009 (9). However, among hospitalized patients, the incidence of hMPV-related respiratory infections can be as high as 6.24% (10). Understanding the impact of hMPV is critical for its prevention and treatment, particularly in healthcare settings during peak respiratory virus seasons.

hMPV is a non-segmented, negatively stranded RNA with a genome of approximately 13 kb comprising eight genes (N, P, M, F, M2, SH, G, and L) (11). It encodes nine proteins, including three surface glycoproteins: fusion (F), small hydrophobic (SH), and glycol (G). The F protein mediates viral adhesion and fusion to host cells via integrins and glycosaminoglycans, serving as a key target for neutralizing antibodies (12). The SH protein suppresses host innate immune responses by inhibiting NF-κB activation and may affect viral fusion (13). The G protein facilitates infection by binding to host glycosaminoglycans and inhibiting RIG-I-dependent gene transcription, further dampening host immune responses (14). Beyond variability in the frequency of detection in recent years, hMPV exhibits a wide genetic variability, particularly within the G gene, which shows nucleotide and amino acid homologies of approximately 56.3 and 34.2%, respectively (9), suggesting the presence of a wide spectrum of viral variants. Conversely, the F gene demonstrates low genetic diversity, indicating a stable evolutionary pattern. hMPV comprises a single serotype with two genotypes, A and B, further subdivided into six lineages (A1, A2a, A2b, A2c, B1, and B2), primarily based on variations within the G gene (15). In addition, the simultaneous circulation of strains of different hMPV sub-genotypes, including B1, B2, and A2b, within closely located geographical regions has been documented (16), highlighting the potential emergence and spread of different hMPV lineages across various regions. Moreover, our pre-COVID-19 study documented the simultaneous emergence of multiple hMPV genetic lineages in Bulgaria (17). Furthermore, the increased frequency of A2b lineage detection has been significantly associated with greater disease severity (9). These findings suggest that some hMPV lineages may be associated with more severe clinical outcomes, highlighting the need for further investigation of their pathogenic potential and implications for patient treatment. They also highlight the importance of preventive measures, including the development of vaccines for young children, to reduce hMPV-related pediatric hospitalizations and prevent future outbreaks similar to those observed in China in 2024.

Furthermore, improving our understanding of the transmission mechanisms, age-related patterns, and genetic characteristics of hMPV. This study aimed to determine the frequency of circulating hMPV, investigate the influence of seasonal and demographic factors, and characterize viral genetic evolution, focusing on mutations in surface proteins and antigen-binding sites. These analyses may help clarify the relationship between viral genetic characteristics and the clinical severity of respiratory diseases.

## 2 Materials and methods

### 2.1 Study design

Between early 2023 and late November 2025, nasopharyngeal specimens from 6,958 patients presenting with symptoms and signs of respiratory infection were tested at the National Reference Laboratory (NRL) for Influenza and Acute Respiratory Diseases (ARD). Specimens were collected from both outpatients and hospitalized patients, and were submitted by general practitioners, hospitals, and regional centers across all 28 districts of Bulgaria. The study included patients of all ages, ranging from 7 days to 98 years, with a median age of 13 years. The sex distribution was nearly equal, with 50.8% male and 49.2% female participants.

Personal and clinical data were collected from cover letters submitted with the specimens by general practitioners, medical centers, emergency departments, and hospital units nationwide. These letters typically included demographic data (age, sex, and location) and clinical information, such as symptoms of respiratory infections (including fever, headache, diarrhea, runny nose, cough, and shortness of breath). The letters also contained diagnoses such as acute respiratory infections and other infectious diseases, alongside associated with complications such as bronchiolitis, bronchitis, and pneumonia.

The study population included symptomatic patients admitted to health facilities who underwent virological testing based on their clinical presentations, as recommended by clinicians. There were no restrictions regarding age or sex; all individuals presenting with symptoms and undergoing testing were included in the study. The Severe Acute Respiratory Infection (SARI) population comprised hospitalized patients who met the World Health Organization definition of severe acute respiratory infection, namely an acute respiratory infection characterized by a measurable temperature of ≥38 °C (100.4 °F) and a cough, with symptom onsets within the previous 10 days.

### 2.2 Sample collection and transportation

Samples were collected in specialized commercial containers containing viral transport medium (up to 2 mL). Collection was performed either during the patient’s visit to the doctor’s office or within the first 24 h after hospital admission. After collection, the samples were transported under refrigerated conditions to the NRL “Influenza and ARD”. Before transportation, the samples were stored for up to 48 h in a refrigerator or, for longer periods, in a freezer at −80 °C.

### 2.3 Exclusion criteria

Patients who exhibited symptoms not commonly associated with respiratory infections and those who were either unable or unwilling to provide consent were excluded. Furthermore, laboratory samples that did not adhere to the established criteria for the transportation and storage of nasopharyngeal specimens were excluded.

The study protocol was approved by the Ethics Committee of the National Center of Infectious and Parasitic Diseases, Sofia, Bulgaria (NCIPD IRB 00006384) (protocol 7/2023), and informed consent was obtained from all participants or their guardians.

### 2.4 Testing

#### 2.4.1 Extraction and real-time polymerase chain reaction (PCR)

Upon arrival at the laboratory, the samples were extracted using an automated extraction system with an Exi-Prep Dx Viral DNA/RNA kit (Bioneer, Daejeon, Republic of Korea), which isolated both DNA and RNA, resulting in a final eluate volume of 100 μL. After extraction, samples were analyzed using real-time PCR on the same day. If immediate analysis was not possible, samples were stored at −20 °C until analysis was performed.

The samples obtained in the laboratory were routinely tested using real-time multiplex PCR for the presence of SARS-CoV-2, influenza A and B viruses, and eight non-influenza respiratory viral pathogens, including hMPV.

For this purpose, we used primers and probes labeled with different fluorescent markers; their sequences have been reported in our previous publication (18).

Four different mixtures were prepared according to the scheme, including primers and probes, and the multiplex enzyme mixture Applied Biosystems™ TaqMan™ Multiplex Master Mix (Thermo Fisher Scientific, Waltham, MA, USA):

mix1: SARS-CoV-2 + influenza A + influenza B;

mix2: AdV + RSV + PIV1;

mix3: BoV + RV + PIV2;

mix4: hMPV + PIV3.

The multiplex PCR mixture was prepared using Applied Biosystems™ TaqMan™ Multiplex Master Mix (Thermo Fisher Scientific, Waltham, MA, USA). Each run included both positive and negative controls. Positive controls for influenza A and B viruses were provided by IRR (USA), whereas AmpliRun DNA/RNA amplification controls (Vircell, Spain) were used for other viruses. Respiratory pathogens were tested using the QuantStudio™ 5 96-well real-time PCR system (Thermo Fisher Scientific, Waltham, MA, USA). Samples with cut-off cycle (Ct) values <38 were considered positive. For further sequencing of hMPV-positive samples, those with Ct values <30 were selected. The samples were separated and frozen on the same day of PCR testing at −80 °C to preserve genomic integrity for longer periods.

#### 2.4.2 Sequencing and phylogenetic amino acid analysis

- Next-generation sequencing (NGS)

Libraries were prepared using the Illumina RNA Prep with Enrichment workflow and Viral Surveillance Panel v2 (Illumina, San Diego, CA, USA) according to the manufacturer’s instructions. Briefly, total RNA was reverse transcribed using random hexamer priming to generate cDNA, which was subsequently used for library construction with enzymatic fragmentation and adapter ligation (Illumina UD Index adapters, V3). Target enrichment was performed using the VSP v2 hybrid-capture probe panel. Input material consisted of 10–100 ng of total RNA per sample. Library fragment size distribution was assessed using QIAxcel Advanced capillary electrophoresis (Qiagen, Hilden, Germany). Libraries were quantified and normalized using a Qubit 4 fluorometer (Thermo Fisher Scientific, Waltham, MA, USA) with the Quant-iT™ dsDNA High-Sensitivity Assay Kit. Sequencing was performed on an Illumina MiSeq platform using a 600-cycle v3 reagent kit.

- Genomic, phylogenetic, and amino acid analysis

The DRAGEN Microbial Enrichment Plus (DME+) software (version 1.1.1), available on the BaseSpace platform from Illumina (Cambridge, UK), was used to assemble the sequences and extract the FASTA files. Sequencing quality control was performed using the Illumina platform QC pipeline, complemented by FastQC for read quality assessment and SAMtools for coverage analysis. Mean depth and breadth of genome coverage were calculated as part of the standard Illumina QC workflow. The mean genome read depth was 856 × (range: 412 × −1,365×). The genome breadth of coverage at ≥10 × averaged 99.5% (range: 98.9–99.9%) across all analyzed samples. *Q*-scores between 35.67 and 36.62.

hMPV sequences were deposited in the GenBank sequence database (BankIt3056596/PZ029062-PZ029078; BankIt3056619/PZ029079-PZ029097). BLAST searches were conducted across multiple databases to retrieve the references and closely related sequences. Multiple sequence alignments were performed using Geneious Prime (Biomatters Ltd., Auckland, New Zealand). Phylogenetic trees were constructed in Geneious Prime using the Maximum Likelihood method with an appropriate nucleotide substitution model selected based on model testing. The robustness of the inferred tree topology was assessed by bootstrap analysis with 1,000 replicates. Bootstrap support values were calculated to evaluate the confidence of individual nodes and are indicated on the corresponding branches of the phylogenetic tree. Final tree visualization and annotation were further refined using the Interactive Tree Of Life (iTOL) tool. Amino acid analysis was performed using BioEdit (www.mbio.ncsu.edu/BioEdit/BioEdit.html, Accessed January 1, 2026) (RRID: SCR_007361). Bulgarian sequences were aligned with the consensus sequences of hMPV strains of genotypes A (AY297749.1) and B (BFJ168778.1). Variable regions of the amino acid sequences were analyzed for glycosylation sites. The NetNGlyc 1.0 and 4.0.3 servers were used to identify N-glycosylation sites and predict the O-glycosylation sites of GalNAc. Sites with scores exceeding a threshold of 0.5 were classified as glycosylated.

### 2.5 Statistical analysis

Fisher’s exact test and the chi-square test were used to analyze categorical variables. For continuous variables, comparisons were made using the Mann–Whitney *U* test, supported by OriginPro software and GraphPad.^1^ *p*-values < 0.05 were considered statistically significant.

## 3 Results

### 3.1 Patients and distribution of respiratory infections

This study included 4,184 hospitalized patients and 2,774 outpatients. Over the approximately 3-year study period, 51% of the patients had an established respiratory infection. Patients from all age groups were studied, and the distribution of infected patients with at least one respiratory virus according to five age groups was: up to 2 (*n* = 1,169/1,942, 60%), 3–4 (*n* = 547/901, 60%), 5–16 (*n* = 1,332/2,863, 46.2%), 17–64 (*n* = 260/676, 38.5%) and ≥65 (*n* = 202/475, 42%) years.

The proportion of patients with confirmed hMPV was 1.4%, accounting for a total of 97 cases. Mono-infections were more common than co-infections (85.6% versus 14.4% /of 97 cases). Co-infections with hMPV and other respiratory viruses were as follows: rhinovirus (RV), *n* = 7 (50% /of 14 cases); adenovirus (AdV), *n* = 4 (28.7%/of 14 cases); bocavirus (BoV), *n* = 3 (21.4%/of 14 cases); and influenza A (H3N2), *n* = 2 (14.29%/of 14 cases).

### 3.2 Seasonal and age distribution of detected hMPV

**Figure 1:**
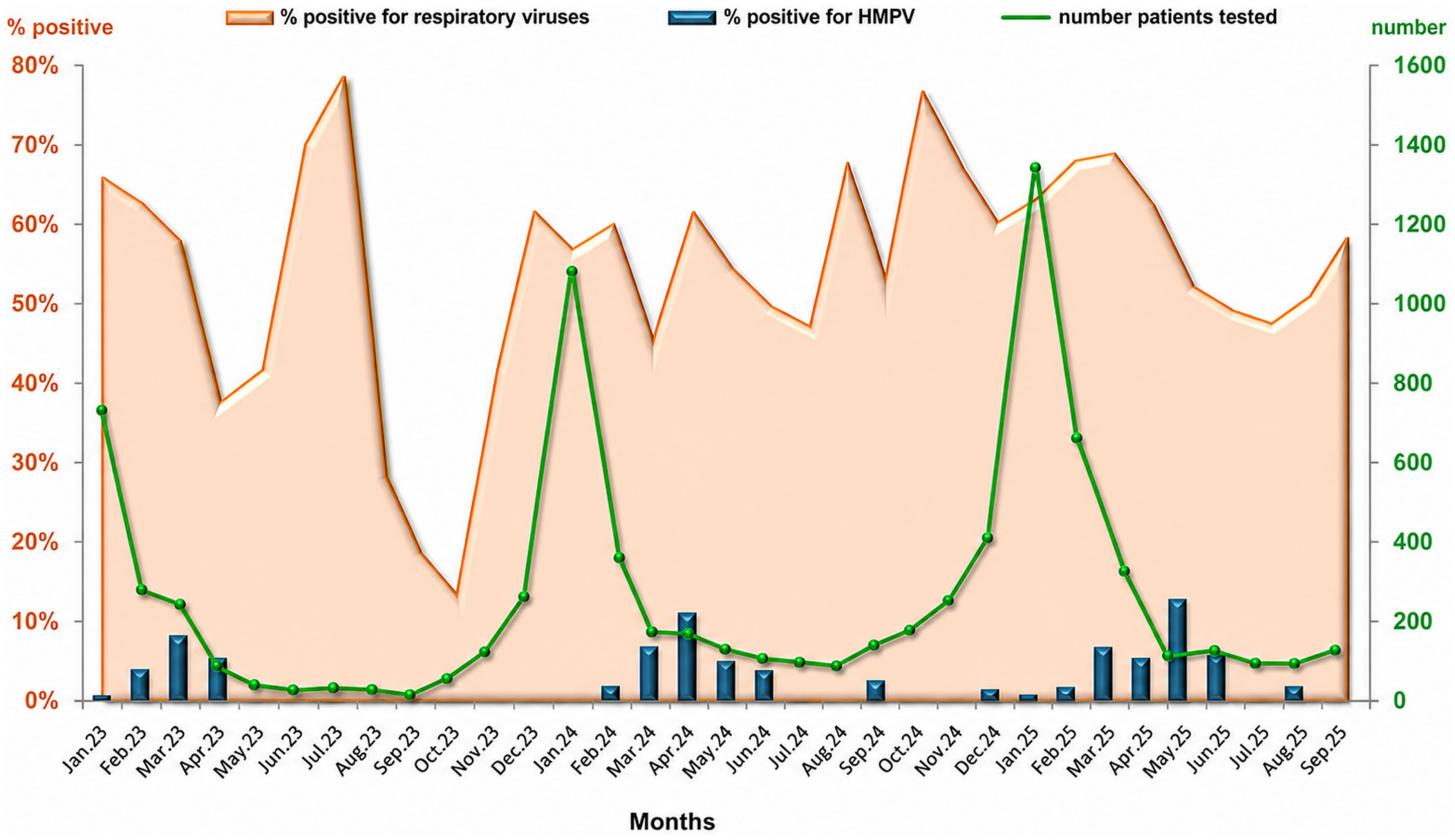
Monthly distribution of detected hMPV from early 2023 to the end of November 2025 in Bulgaria. HMPV, human metapneumovirus.

During the study period, the overall confirmed respiratory viruses were relatively stable across the 3 years: 2023 (49.9%, 799/1,599), 2024 (49.8%, 1,257/2,526), and 2025 (53%, 1,345/2,519). In 2023, the detection rate of hMPV was 14.3%, decreasing to 10.6% in 2024 and 0.9% in 2025. During the winter months (December to late February), the number of confirmed respiratory infections increased significantly. However, the highest hMPV detection rates were observed in the spring, specifically between March and April. In 2023, the peak was observed in March at 6%, whereas in 2024 and 2025, the peak shifted to April, with rates of 8 and 10%, respectively (Figure 1).

**Figure 2:**
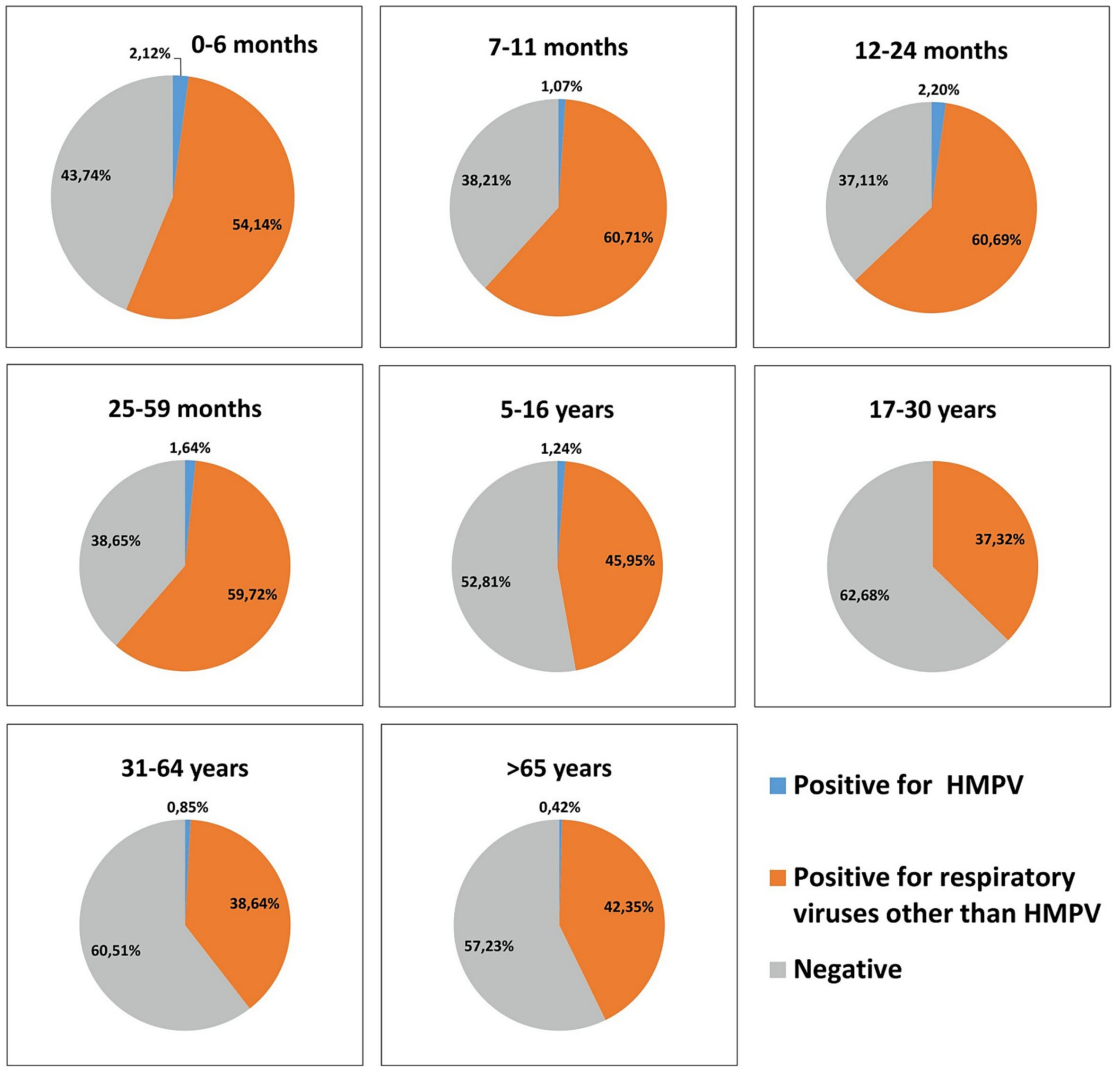
Distribution of detected hMPV across seven age groups: 0–6 months, 7–11 months, 12–24 months, 25–59 months, 5–16 years, 17–30 years, 31–64 years, and >65 years between 2023 and 2025 in Bulgaria. HMPV, human metapneumovirus.

At least one case of hMPV infection was detected in all age groups except those aged 17–30 years (age groups included 0–6 months, 7–11 months, 12–24 months, 25–59 months, 5–16 years, 31–64 years, and ≥65 years) (Figure 2). The median age of patients infected with hMPV was 7.5 ± 12.5 years. A relatively high infection rate was observed among children aged <5 years, reaching 2.8% (*p* < 0.05). Within this age group, infants aged 0–6 months and children aged 1–2 years exhibited the highest rates, both at 2.2%, significantly higher than those of individuals aged >65 years (0.4%, *p* < 0.05).

**Figure 3:**
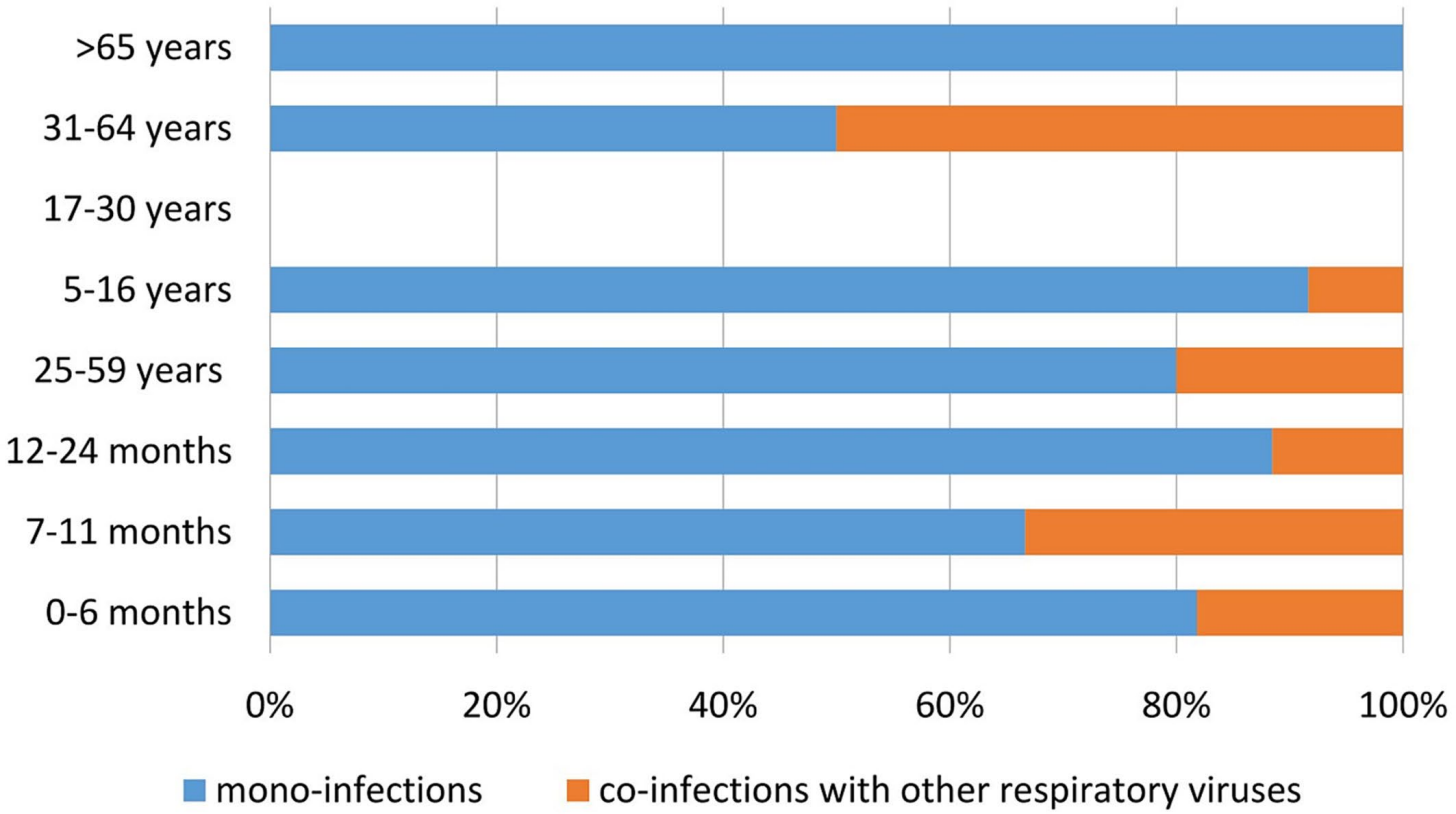
Distribution of the proportion of mono- and co-infections of hMPV with other respiratory viruses across seven age groups: 0–6 months, 7–11 months, 12–24 months, 25–59 months, 5–16 years, 17–30 years, 31–64 years, and >65 years, between 2023 and 2025 in Bulgaria. HMPV, human metapneumovirus.

Mono-infections, including hMPV, were more frequent in children aged <5 years than in older age groups; this age group accounted for 47% of all mono-infections and 64% of co-infections. Across age groups, the proportion of mono-infections generally exceeded that of co-infections, with the exception of adults aged 31–64 years, where mono- and co-infections occurred at similar rates. Notably, no co-infections involving hMPV were detected in patients aged ≥65 years (Figure 3).

### 3.3 Genotyping and phylogenetic analysis

**Figure 4:**
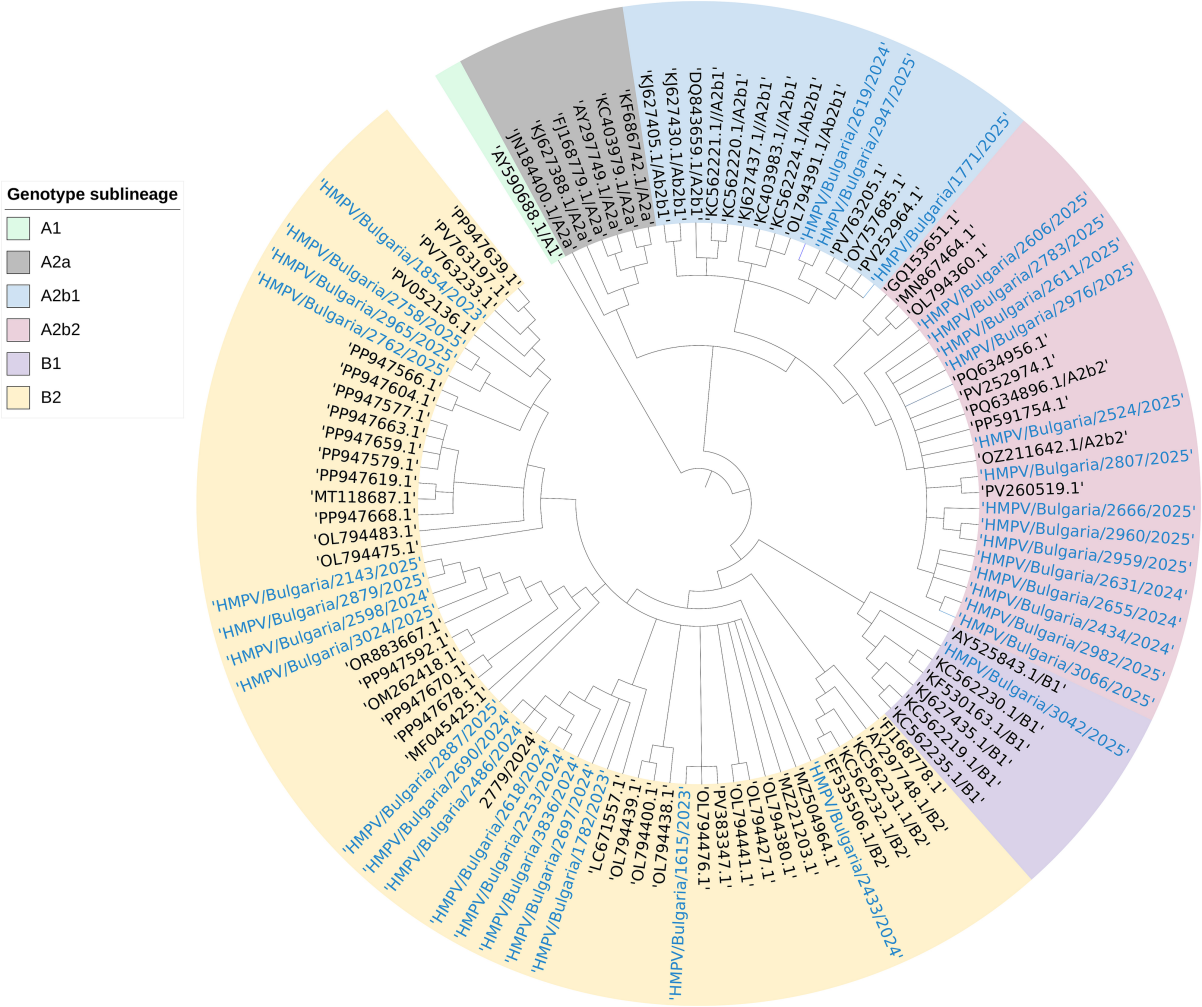
Phylogenetic analysis of hMPV, in which a tree was constructed based on the G and F proteins and the entire viral genome. Genetic distances were measured according to the Jukes-Cantor model. The phylogenetic tree was constructed using the neighbor-joining algorithm in Geneious Tree Builder. Sequences of reference strains representative of known genotypes were obtained from GenBank with the corresponding accession numbers. Bulgarian sequences are presented in blue. The tree was rooted based on the sequence of avian metapneumovirus submitted by a research team in the USA in 2004 (AY590688.1/A1). HMPV, human metapneumovirus.

Over the approximately 3-year study period, а total of 50/97 (51.5%) samples with Ct values ≤ 30 were positive for hMPV, of which 37/50 (74.0%) samples with sufficient genome integrity were successfully sequenced. The remaining samples could not be sequenced because of low viral loads. Reference strains for each genotype (A and B) were selected through BLAST searches and are indicated in the phylogenetic tree (Figure 4), along with closely related sequences from other countries.

Phylogenetic analysis revealed similar nationwide proportions of genotypes A and B, accounting for 40.5% (15/37) and 51.3% (19/37), respectively. The most frequently identified subclade was B2 (19 samples, 41.3%), followed by A2b (14 samples, 37.8%). The smallest proportions were found in subclades A2b1 (3 samples, 8.1%) and B1 (1 sample, 2.7%). Strains collected in 2025 showed the greatest subclade diversity, including B1, B2, A2b2, and A2b1, whereas all 2023 strains belonged exclusively to B2. In 2024, B2 accounted for 69% (9/13) of the samples, declining to 33% (7/21) in 2025, when clade A2b2 predominated at 52% (11/21).

### 3.4 Amino acid analyses

#### 3.4.1 Fusion (F) protein

The amino acid sequences of the F-proteins from 37 Bulgarian hMPVs were aligned, excised, and compared with those of prototype hMPV strains of genotypes A (AY297749.1) and B (BFJ168778.1). Nine amino acid substitutions were detected in genotype A: two in the F2 subunit (positions 61 and 76), and seven in the F1 subunit (positions 143–518) (Table 1). The T143K and I449V substitutions were localized in the HRA and HRB domains, respectively. Genotype B showed a higher substitution rate: 72% (24/33). Two amino acid substitutions occurred in F2 (positions 36 and 64), and 22 in F1 (positions 142–528) (Table 1). Of these, K142R, K143R, and E167D were located in the HRA region; S444N and D475E in the HRB region; and N528S in the TM region.

**Table 1:** Amino acid substitutions were identified in the F proteins of human metapneumovirus (HMPV) genotypes A and B circulating in Bulgaria during the 2023–2025 season.

Genotype	Amino acid site substitutions	Sublineage A2b1 frequency (%, *n* = 3)	Sublineage A2b2 frequency (%, *n* = 14)
A, *n* = 17	S61A	3 (100)	14 (100)
	K76N		2 (14)
	T143K	3 (100)	14 (100)
	K171R		1 (71)
	G294E	3 (100)	14 (100)
	I449V	3 (100)	14 (100)
	I510V	3 (100)	
	F511L	1 (33)	
	K518R		14 (100)

Genotype	Amino acid site substitutions	Sublineage B1 frequency (%, *n* = 1)	Sublineage B2 frequency (%, *n* = 19)
B, *n* = 20	L36H		3 (15)
	P64S		3 (15)
	K142R		1 (5)
	K143R	1 (100)	
	E167D		6 (31)
	K179R	1 (100)	4 (21)
	T223N		19 (100)
	V231I		2 (10)
	D280N		19 (100)
	D296N	1 (100)	
	G308V		1 (5)
	I392T		17 (89)
	R396Q		19 (100)
	Q413R		5 (26)
	S444N		19 (100)
	D475E	1 (100)	
	I498V	1 (100)	
	V500A	1 (100)	
	L503S		1 (5)
	I513T		1 (5)
	A517T		1 (5)
	T521A		19 (100)
	A523T		8 (42)
	N528S		4 (21)

Distribution of substitutions in the F protein of Bulgarian strains relative to the reference sequences: AY297749.1 for genotype A and FJ168778. The distribution of these substitutions is categorized by their prevalence in the following sublineage/clades: A2b1, A2b2, B1, and B2.

Several substitutions were observed within antigenic regions (epitopes) (19): 2 (residues 132 and 152) for genotype A, T143K; for genotype B, K142R and K143R. In genotype B, other substitutions were also identified in antigenic regions 3 (residues 177–179) and 5–6 (residues 386–397), in one region, K179R, and in the other, I392T and R396Q. No substitutions were identified in any of the Bulgarian sequences in antigenic region 4 (residue 245). All analyzed Bulgarian sequences had three potential N-linked glycosylation sites at positions 57 (NLT), 172 (NLT), and 353 (NIS); the latter located in antigenic regions 5–6 (residues 386–397).

#### 3.4.2 Glycoprotein (G) protein

**Figure 5:**
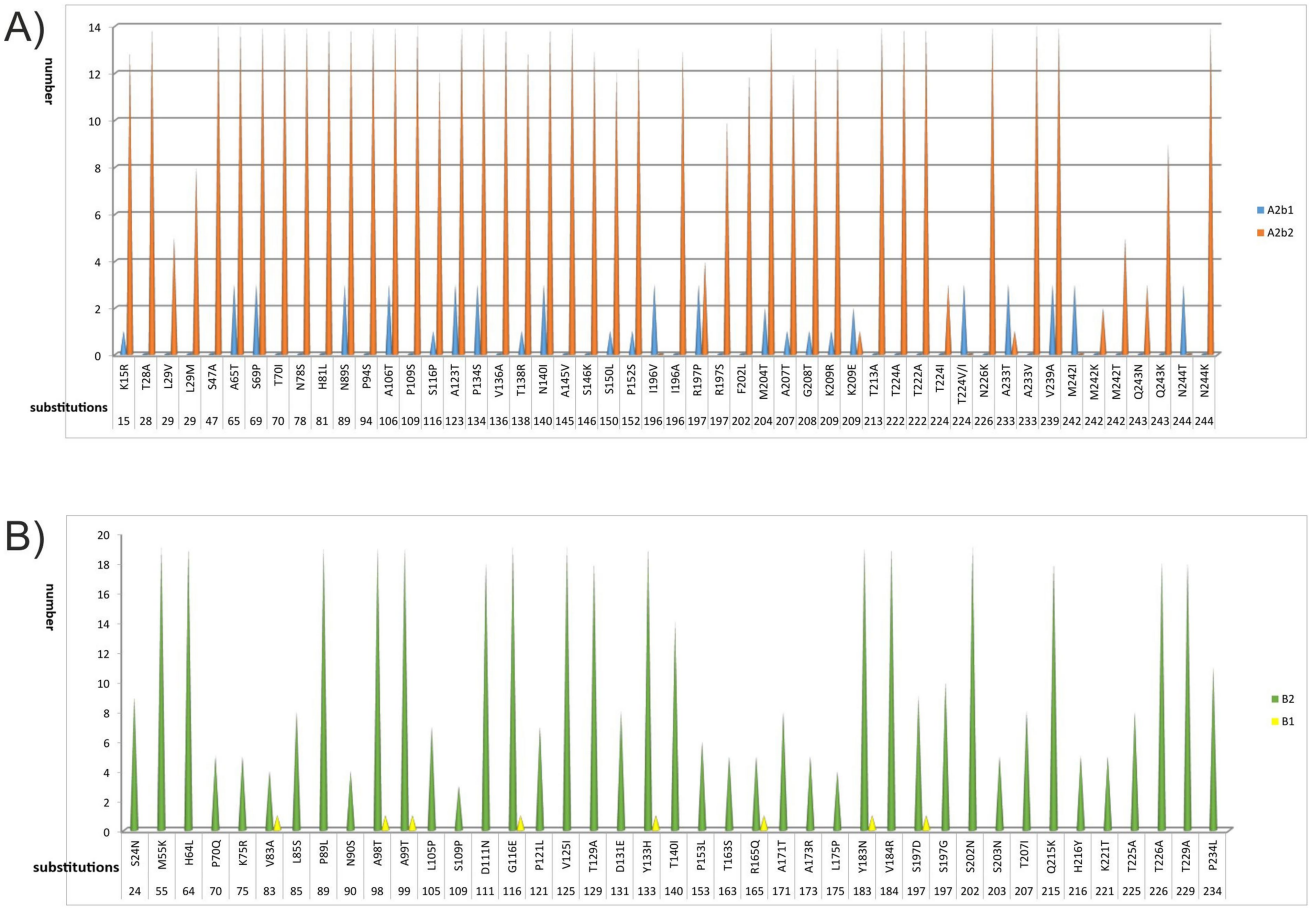
Amino acid substitutions identified in the G proteins of human metapneumovirus: genotypes 5A **(A)** and 5B **(B)** circulating in Bulgaria during the 2023–2025 season. Distribution of substitutions in the G protein of Bulgarian strains relative to the reference sequences: AY297749.1 for genotype A and FJ168778.1 for genotype B. Distribution of these substitutions in the following genetic lineages: A2b1, A2b2, B1, and B2.

The amino acid sequences of the G proteins from all Bulgarian hMPV were aligned, trimmed, and compared with those of the prototype hMPV strains of genotypes A (AY297749.1) and B (FJ168778.1). Fifty substitutions were identified in genotype A sequences, and 54 in genotype B (Figures 5A,B). Two N-glycosylation sites were identified in all Bulgarian hMPV genotype A sequences at positions 18 (NAS) and 40 (NYT). In contrast to the reference strains, all Bulgarian sequences showed the loss of an N-glycosylation site at position 89 (NPT), where asparagine (ASN) was replaced by serine (SER), resulting in an S89N (SXT) substitution. For genotype B, two N-glycosylation sites were found in all sequences: 30 (NAT) and 176 (NTT). Furthermore, in all clade B2 sequences, potential N-glycosylation sites were observed at positions 58 (NMT) and 179 (NQT). However, in clade B1 sequences, these two sites were lost because of substitutions at positions T60I (leading to NMI) and T181I (leading to NQI). Conversely, clade B1 sequences exhibited new N-glycosylation sites compared with clade B2 at positions 101 (NST) and 183 (NAS), associated with D101N, N183Y, and R185S substitutions.

**Figure 6:**
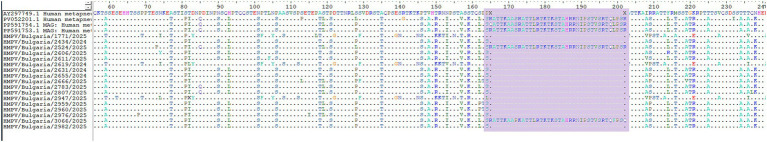
Deduced amino acid alignment of the G protein in hMPV A2b strains is presented relative to the sequence of the classical non-duplication prototype A2b (GenBank accession number AY297749.1). Identical residues are indicated by dots. Outlined rectangles visualize duplications (111 np-dp) or 37 amino acids in a region of the hMPV strains isolated in Bulgaria and other countries. HMPV, human metapneumovirus.

Alignment with the reference strain A2b AY297749.1 revealed a duplication of the 111 nt-dup motif (duplicated in the 458–568 bp region of the G gene) in two Bulgarian A2b2 strains (11% of genotype A, *n* = 17), similar to those found in other countries (Figure 6). This duplication creates an additional 13 potential acceptor sites for O-linked sugars, increasing the total to 80. The G protein of classical A2b2 strains without the 111 nt-dup duplication had 65–67 potential acceptor sites for O-linked sugars, while A2b1 strains had 69–71 (Table 2). Compared with reference strain FJ168778.1, the Bulgarian B2 strains had five newly emerged potential acceptor sites for O-linked sugars, while the Bulgarian B1 strains gained nine (see Table 2).

**Table 2:** The distribution of new potential acceptor sites for O-linked sugars resulted from amino acid substitutions detected in the G proteins of human metapneumovirus (HMPV) genotypes A and B, which circulated in Bulgaria during the 2023–2025 season.

Amino acid site substitutions	Amino acid site	Sublineage (A2b1), number frequency (%, *n* = 3)	Sublineage (A2b2), number frequency (%, *n* = 14)
P57T	57	1 (33)	
A65T	65	3 (100)	14 (100)
N78S	78		14 (100)
N89S	89	3 (100)	14 (100)
P94S	94		14 (100)
A96S	96	3 (100)	
P100S	100	2 (66)	
A106T	106	3 (100)	14 (100)
P109S	109		14 (100)
A123T	123	3 (100)	14 (100)
P134S	134	3 (100)	14 (100)
N140T	140	3 (100)	
A145T	145	3 (100)	
A191T	191	3 (100)	
R197S	197		10 (71)
R198S	198	3 (100)	
A199T	199	3 (100)	
M204T	204	3 (100)	14 (100)
G208T	208	3 (100)	14 (100)

Amino acid substitutions	Amino acid site	Sublineage (B1), number frequency (%, *n* = 1)	Sublineage (B2), number frequency (%, *n* = 19)
A82T	82	1 (100)	
A98T	98	1 (100)	19 (100)
A99T	99	1 (100)	19 (100)
P153T	153	1 (100)	
A171T	171		8 (42)
R185S	185	1 (100)	
A187T	187	1 (100)	
I188A	188	1 (100)	
A192T	192		3 (16)
T198A	198	1 (100)	
Q215T, S	215	1 (100)	1 (5)

These changes were analyzed in relation to the HMPV genetic lineages: A2b1, A2b2, B1, and B2. Comparisons were made with the amino acid sequences of the G proteins from the reference strains of genotypes A (AY297749.1) and B (FJ168778.1).

### 3.5 Clinical characteristics of the studied patients

In this study, which included 97 patients who tested positive for hMPV, clinical information was derived from accompanying medical documentation submitted with the specimens, and symptom reporting was available for a subset of patients (56%). The most commonly observed symptoms were cough (50%, 22 patients), fever (38%, 20 patients), rhinitis (21.2%, 11 patients), vomiting (15.4%, 8 patients), diarrhea (13.5%, 7 patients), shortness of breath (1.9%, 1 patient), and fatigue (1.9%, 1 patient). During the study period, diagnostic information was available for 75% of patients who tested positive for human metapneumovirus (hMPV). Among these patients, 34% (32 of 94) had upper respiratory tract infections, and 55% (52 of 94) had lower respiratory tract infections (LRTs). The most common complications affecting LRTs among patients infected with hMPV were bronchiolitis, observed in 46% (24 of 52) of cases, and pneumonia, which occurred in 30.7% (16 of 52) of cases. In some cases, more than one lower respiratory tract diagnosis was recorded. Further, a total of 42 (43%) patients were hospitalized, and 55 (56.7%) received outpatient or emergency care or were managed without hospital admission. Of the hospitalized hMPV-infected patients, eight (19%) met the criteria for SARI, indicating a notable proportion of severe disease among hospitalized cases. In comparison, 4,016 hospitalized patients tested positive for at least one respiratory virus other than hMPV, with 293 (7.3%) of them classified as having SARI (*p* = 0.0107). This comparison suggests a higher proportion of SARI among hospitalized hMPV-infected patients; however, this observation should be interpreted cautiously, as the analysis was not adjusted for potential confounding factors such as age, comorbidities, or testing indications.

### 3.6 Clinical manifestation and genetic characterization of hMPV

A correlation was found between the age distribution of pediatric patients and the genetic distribution of hMPV, particularly its genotypes, subgenotypes, and clades, as well as the presence of features such as genomic duplications or mixed infections. These factors were further examined in relation to the likelihood of upper and lower respiratory tract complications (Table 3).

**Table 3:** The distribution of identified human metapneumovirus genetic lineages and clades categorized based on whether they cause upper or lower respiratory tract complications in children in two groups: those under 5 years of age and those up to 16 years of age.

Factor	<5 years URTI (*n* = 31)	<5 years LRTI (*n* = 14)	*p*-value	5–16 years URTI (*n* = 14)	5–16 years LRTI (*n* = 18)	*p*-value
Genotype						
A, *n* (%)	3 (9)	7 (50)	0.0053	2 (14)	6 (33)	0.4123
B, *n* (%)	6 (19)	5 (36)	0.2771	1 (7)	4 (22)	0.3547
Sublineage/clades						
A2B1, *n* (%)	0 (0)	1 (7)	0.3111	0 (0)	2 (11)	0.1301
A2B2, *n* (%)	3 (9)	6 (42)	0.0171	2 (14)	6 (33)	0.4123
B1, *n* (%)	0 (0)	0	n.s	0 (0)	0 (0)	
B2, *n* (%)	6 (19)	5 (35.7)	0.2771	1 (7)	4 (22)	0.3547
Duplication						
111 nt-dup, *n* (%)	0 (0)	2 (14)	0.0919	0 (0)	0 (0)	n.s*
Viral co-infection, *n* (%)	4 (12)	2 (14)	n.s	0 (0)	1 (5)	n.s

n.s*, non-significant.

This study found that children under 5 years of age infected with hMPV genotype A were more likely to develop lower respiratory tract complications than upper respiratory tract complications (*p* < 0.05), as presented in Table 3. Notably, clade A2b2 was associated with a higher incidence of lower respiratory tract complications (*p* < 0.05). Patients infected with hMPV genotype B were at a higher risk of developing SARI (*p* < 0.05; Table 4). This association was observed particularly among patients with lower Ct values, consistent with higher viral load. Differences in clinical presentation between specific sublineages were explored, and studies have suggested that A2b with the duplication present may lead to lower respiratory tract complications (20). However, this study did not confirm these findings as only two strains with A2b duplications were identified, making it insufficient for broad generalization. Overall, the observed genotype–clinical associations suggest potential differences in clinical expression between viral lineages; however, the modest number of severe cases and incomplete availability of detailed clinical severity indicators limit definitive conclusions regarding genotype-specific pathogenicity.

**Table 4:** The distribution of factors includes viral load, median age, and various age groups: 0–6 months, 7–11 months, 12–24 months, 5–16 years, 17–30 years, 31–64 years, and those over 65 years.

Factor	SARI (*n* = 8)	non-SARI (89)	*p*-value
Viral load/Ct, mean (±SD)	26 (4.8)	30 (5.1)	*p* = 0.018
Age mean (±SD)	3 (2.5)	7.9 (13)	n.s.*
Age group			
0–6 months	0 (0)	12 (13)	0.5902
7–11 months	1 (13)	2 (2)	0.2298
12–24 months	4 (50)	20 (22)	0.1009
25–59 months	1 (13)	14 (15)	n.s.
5–16 years	2 (25)	33 (37)	0.7069
17–30 years	0 (0)	0 (0)	n.s.
31–64 years	0 (0)	4 (4)	n.s.
>65 years	0 (0)	2 (2)	n.s.
Sex			
Female	5 (63)	40 (44)	0.4657
Male	3 (38)	47 (52)	0.4780
Viral co-infection	1 (13)	13 (14)	n.s.
Genotype			
A	2 (25)	15 (16)	0.6263
B	4 (50)	16 (18)	0.0540
Sublineage			
A2B1	0 (0)	3 (3)	n.s.
A2B2	2 (25)	12 (13)	0.3249
B1	1 (13)	0 (0)	0.0825
B2	3 (38)	16 (18)	0.1865
Duplication			
111 nt-dup, *n* (%)	0 (0)	2 (2)	n.s.

n.s*, non-significant.

Gender and the presence of co-infections with other respiratory viruses, as well as genetic characteristics, are also considered. This includes genetic groups A and B, along with genetic lineages or clades A2b1, A2b2, B1, and B2. Additionally, the presence of a characteristic 111-nucleotide duplication is taken into account. These factors are categorized based on their presence in patients with severe acute respiratory infection (SARI) or in those without.

## 4 Discussion

Recent trends in the unusual increase of certain respiratory pathogens highlight the need for continued monitoring of genetic variability, virulence, and clinical severity. This trend includes the emergence of SARS-CoV-2 in 2020 and the subsequent evolution of different variants over time, resulting in different clinical severities of COVID-19 (21). In addition, a significant increase in hMPV infections was reported in China in 2024 (4), alongside an increase in influenza cases at the beginning of the 2025–2026 influenza season in England. This surge was associated with the emergence of a new clade “K” of the H3N2 virus.^2^ Given the absence of extensive studies in Bulgaria following the COVID-19 pandemic regarding the spread and genetic traits of hMPV, we conducted a 3-year investigation to clarify its clinical and genetic characteristics. We built on our previous study using whole-genome sequencing, which allowed us to cover significant coding regions of the genome and analyze important surface proteins (17). Further, we made important connections between the predisposition to develop complications in certain species and the genetic predisposition to hMPV. This study found a low prevalence of hMPV in Bulgaria during the 3 years following the COVID-19 pandemic. Data from 2003 to 2023 indicate a prevalence rate in China ranging from 0.97 to 15.88% (8). A large-scale study reported an infection rate of 3.3% due to hMPV infections in 2024. (22). Other studies confirm our seasonal pattern, indicating higher prevalence during the spring and winter months (23). Research shows that individuals presenting with acute respiratory tract infections in spring have an approximately nine-fold higher risk of hMPV infection than those sampled in summer and fall (8).

The pattern of hMPV prevalence has changed since the COVID-19 pandemic. The mean age of prevalence increased from 2.3 ± 2.0 years pre-pandemic to 3.6 ± 2.5 years in 2023 (24). This shift indicates an altered epidemiology, as lockdowns left many children unexposed to respiratory viruses, increasing susceptibility among older preschoolers. Increased testing of older children may also contribute to a higher mean age at diagnosis (25). Globally, hMPV accounts for 5–15% of respiratory infections, with peak incidence observed in infants aged 6 to 12 months. However, in this study, we reported peak rates at ages 1–2 years, with a proportion of 2.2% (26). Children aged 0–4 years represent the largest group of cases of hMPV infection (27). Other studies further confirmed these findings, revealing a significant difference in the prevalence of the virus between individuals aged <5 years and adult patients (*p* < 0.001) (28). Another study confirmed that the prevalence of hMPV was highest among children aged 0–6 years, although higher levels of RSV were observed in the same age group. Similar prevalence rates of parainfluenza virus type 3 and influenza have been reported in other age groups (29). Furthermore, a study conducted in India found that children aged 1–2 years consistently showed the highest levels of hMPV positivity both before and after the COVID-19 pandemic, reflecting the increased incidence of hMPV infections in this age group, as reported in this study (22). The epidemiological changes in hMPV after the pandemic are also reflected in an increase in co-infections and a modified age distribution, which accounts for the slight increase in detected co-infections from 13 to 14.4% compared to our previous study (17). A separate study found that co-infection with other respiratory viruses is common among children infected with hMPV (15–35%) (30). However, our study reported a lower rate of co-infection. Notably, children aged <5 years have a higher incidence of co-infection than older children (31). Furthermore, another study showed that rhinovirus, adenovirus, and influenza A are the four co-pathogens of hMPV, consistent with our findings (32).

Contrary to the observed changes in age patterns following the COVID-19 pandemic, no significant changes in genetic diversity were observed in a previous study conducted between 2016 and 2019 (17). We identified two genotypes, A and B, along with three genetic lineages, A2b, B1, and B2. A separate study conducted in China between 2017 and 2023 reported higher diversity of the five sub-genotypes/clades (8). Recent studies identified three new hMPV subtypes: A2c, A2b1, and A2b2. However, the phylogenetic relationships between these subtypes are not universally accepted. In this study, we adhered to the 2012 classification, which divided the subtype A2b into A2b1 and A2b2 (12). This classification has been supported by other publications since 2020, as well as by the classification proposed by Nextclade v3.18.1 (https://clades.nextstrain.org/, Accessed January 1, 2026) (33, 34). Molecular analyses indicate that A2b1 and A2b2 diverged approximately a decade after the split of A2 into A2a and A2b (12). Recent studies indicate that the most common genotype A of hMPV is A2b2, whereas B2 is the most prevalent genotype B, consistent with our findings (34, 35). Recent data show that B2 strains often alternate with A2 strains in 2-year cycles, suggesting a resurgence of B2 in some regions by 2024 (36). In 2025, we predicted a global increase in the prevalence of clade A2b2, as confirmed in this study (https://nextstrain.org/blog/2025-01-09-human-metapneumovirus-analysis-and-resourcesy, Accessed January 1, 2026).

The F protein is highly immunogenic and can induce protective immunity, making it a potential target for vaccines, monoclonal antibodies, and antiviral drugs. Amino acid changes in the antigenic sites of the F protein of hMPV may allow the virus to evade immunity developed from previous infections (37). Although the F gene/protein is relatively conserved, it possesses sufficient variability to discriminate between genotypes, consistent with our findings (38). In this study, we identified deletions in three antigen-binding sites and the appearance of N-linked glycosylation sites in domains 4–6 that were not detected in our previous analyses. In our study, we identified substitutions at two critical sites, specifically in the HRA and HRB domains, which are essential for protein folding during fusion (39). Furthermore, we confirmed a trend from our previous studies showing that genotype B has a higher number of amino acid substitutions than genotype A2b. Notably, we observed an increase in substitutions for genotype A, from 2 to 9, and for genotype B, from 11 to 24, compared with our analysis of Bulgarian strains from 2016 to 2019 (17). We identified two N-linked glycosylation sites in genotype A and three in genotype B, all of which were located at the antigenic site. The F protein has various N-linked glycosylation sites, such as N57, N172, and N353, which were confirmed in this study (40). Overall, the gradual accumulation of amino acid changes and N-linked glycosylation in the F protein of hMPV are key mechanisms employed by the virus to evade preexisting immunity (41).

In our previous study (17) of the hMPV genome, we focused mainly on the structure of the F protein, which can be noted as a gap in our research. In this study, we aimed to provide a more comprehensive understanding of the genetic variability and evolutionary dynamics of this pathogen by analyzing the amino acid sequences of the F and G proteins.

Тhe G protein of hMPV shows a high degree of sequence constancy within lineages but considerable variability between them. The analysis revealed significant genetic diversity among the hMPV isolates, with amino acid substitutions specific to certain genotypes, which is consistent with previous studies. Glycosylation influences the properties of glycoproteins by affecting their folding, stability, resistance to proteolysis, and intracellular trafficking. They also play a critical role in viral infectivity and immune evasion by masking epitopes (42). In this study, we predicted a relatively large number of potential O-linked glycosylation sites, 65–71, in non-duplicated site sequences, whereas only a few N-linked 2 sites were identified in the analyzed isolates. This finding is consistent with other studies that have shown that hMPV has 2–5 predicted N-linked sites and 60–66 predicted O-linked sites (35). N-Linked glycosylation is genotype-specific (43).

Duplications of up to 180 nucleotides in the G gene are common in the genotype A2b. Notably, a similar evolutionary process occurred in RSV, in which two independent duplications occurred in the G gene. Strains with these duplications quickly became dominant RSV subtypes. Both RSV and hMPV have shown that duplication of the G gene can provide an evolutionary advantage (44, 45). hMPV-A2b180nt-dup strain emerged between 2011 and 2013 (20), and a case of the A2b111nt-dup strain was reported in 2017 (46). In our study, we only detected the A2b111nt-dup strain, which accounted for 11% of our findings, which is consistent with other research efforts (34). Conversely, the hMPV-A2b180nt-dup strain was not detected in our study; however, it was noted in another large-scale study, where it accounted for a larger percentage of 12.9% of all A sequences based on 1,462 reports from 37 countries (34).

Data from 2023 show that hospitalized infants had the highest number of reported cases of hMPV in the last 6 years (36). This trend, together with the high incidence of hMPV infections among infants found among Bulgarian children, suggests that hMPV may become a leading cause of hospitalization and child mortality. A study has shown that infants under 1 year of age face a risk of severe illness from hMPV, which is comparable to the risks associated with RSV and influenza (47). Studies have shown that hMPV is a major cause of lower respiratory tract complications in children aged <5 years, often leading to bronchiolitis and pneumonia (48). Furthermore, this study showed that hMPV infection is a significant cause of SARI in hospitalized patients. In an earlier study, hMPV was identified as the leading cause of SARI among 17 other respiratory viruses (49).

The clinical findings in our cohort provide important context for interpreting the genotype-specific results. Lower respiratory tract involvement was common, particularly among young children, and a notable proportion of hospitalized patients met the criteria for SARI, indicating clinically significant disease. Within this framework, the observed associations between viral genetic characteristics and disease manifestation suggest that differences in circulating lineages may contribute to variability in clinical presentation. However, given the limited number of severe cases and variability in available clinical detail, these findings should be interpreted with caution. This study also showed that the A2b2 strain of hMPV was associated with a higher incidence of lower respiratory tract complications in children aged <5 years. Patients infected with hMPV genotype B are at an increased risk of SARI, which is accompanied by a higher mean viral load. The B2 strain causes similar symptoms in both the upper and lower respiratory tracts. Conversely, the A2b strain is often associated with more severe respiratory infections, and studies have suggested that A2b with the duplication present may lead to lower respiratory tract complications (20). However, this study did not confirm these findings as only two strains with A2b duplications were identified, making it insufficient for broad generalization. In addition, the limited number of successfully sequenced samples was mainly due to the low viral load in a significant proportion of cases, which reduced sequencing efficiency and genome completeness. We also recognize that the lack of detailed clinical data, such as specific clinical and laboratory findings and length of hospital stay, limits our ability to draw more in-depth conclusions regarding severe hMPV infections. Addressing these limitations will be the focus of future research.

In conclusion, this study identified hMPV as a significant respiratory pathogen that causes tract infections in infants. We found that В genotype of hMPV is a major contributing factor to the development of SARI in children. Recent years have revealed genetic diversity among established hMPV genotypes, with an increase in mutations in the G and F proteins, especially in regions that are important for evading immune defenses. We hypothesized that genotype A2B, among the different genetic lineages, contributes significantly to an increased risk of developing lower respiratory tract complications. Notably, this genotype has become predominant in Bulgaria and other countries in the last year (2025), along with a higher clinical severity of respiratory diseases caused by hMPV, especially in infants. Taken together, these findings highlight the clinical burden of hMPV infection in young children and underscore the need for continued efforts toward preventive strategies, including vaccine development for high-risk pediatric populations.

## Data Availability

The datasets presented in this study can be found in online repositories. The names of the repository/repositories and accession number(s) can be found in the article/supplementary material.

## Footnotes

1
https://www.graphpad.com/quickcalcs/contingency1

2
https://www.who.int/emergencies/disease-outbreak-news/item/2025-DON586
